# The Role of MAO-B Inhibitors in Fatigue in Parkinson’s Disease: A Narrative Review

**DOI:** 10.3390/jcm14082598

**Published:** 2025-04-10

**Authors:** Silvia Galli, Pierre Pacilio, Edoardo Bianchini, Marika Alborghetti, Lanfranco De Carolis, Pietro Lombardo, Francesco Garramone, Marco Salvetti, Domiziana Rinaldi

**Affiliations:** 1 Department of Neuroscience, Mental Health and Sensory Organs (NESMOS), Sapienza University of Rome, Via di Grottarossa, 1035, 00189 Rome, Italy; silvia.galli@uniroma1.it (S.G.); pierre.pacilio@uniroma1.it (P.P.); edoardo.bianchini@uniroma1.it (E.B.); marika.alborghetti@uniroma1.it (M.A.); lanfranco.decarolis@uniroma1.it (L.D.C.); pietro.lombardo@uniroma1.it (P.L.); francesco.garramone@uniroma1.it (F.G.); marco.salvetti@uniroma1.it (M.S.); 2 AGEIS (Autonomy, Gerontology, E-Health, Imaging & Society Unité de Recherche), Université Grenoble Alpes, 38000 Grenoble, France; 3 Istituto di Ricovero e Cura a Carattere Scientifico, Istituto Neurologico Mediterraneo Neuromed, 86077 Pozzilli, Italy

**Keywords:** Parkinson’s disease, fatigue, MAO-B inhibitors, selegiline, rasagiline, safinamide

## Abstract

**Background:** Fatigue is a common and debilitating non-motor symptom (NMS) in Parkinson’s disease (PD), significantly affecting patients’ quality of life. MAO-B inhibitors are effective therapy for motor symptoms and fluctuations and may also play a role in fatigue management. **Methods:** We searched PubMed for English-language articles (January 1978–August 2024) using keywords including “selegiline”, “rasagiline”, “safinamide”, “MAO-B”, “fatigue”, and “Parkinson’s disease”. Clinical trials, observational, and preclinical studies were included. **Results:** While the role of MAO-B inhibitors in fatigue remains unclear, evidence suggests potential benefits. Selegiline has shown effectiveness in improving fatigue in animal models, supporting its potential utility in treating fatigue and motivational impairments in PD patients. Rasagiline has been associated with reduced fatigue progression in early PD, with some studies showing significant improvements compared to placebo. Safinamide, with its dual action as an MAO-B inhibitor and glutamate modulator, may further enhance fatigue management. Its ability to reduce glutamate release is particularly relevant, given the role of glutamate overactivity in PD-related fatigue. Studies indicate safinamide can significantly reduce fatigue levels. **Conclusions:** Fatigue in PD is a complex symptom with multiple contributing factors. While MAO-B inhibitors may support fatigue management, their precise role and optimal use require further investigation.

## 1. Introduction

Parkinson’s disease (PD) is a chronic, progressive neurodegenerative disorder primarily characterized by motor symptoms such as bradykinesia, resting tremor, and rigidity [1]. These manifestations arise from the nigrostriatal degeneration of dopaminergic neurons, resulting in dopamine deficiency [2]. While motor symptoms are the clinical hallmark of PD, the widespread neurodegeneration affecting multiple neurotransmitter systems contributes to a broad spectrum of non-motor symptoms (NMS), which significantly affect the quality of life (QoL) of PD patients, especially in the advanced stages [3].

NMS in PD encompasses a wide range of dysfunctions, including autonomic disturbances (e.g., orthostatic hypotension, urinary dysfunction, constipation), neuropsychiatric symptoms (e.g., depression, anxiety, apathy, psychosis), cognitive impairment, sleep disorders, sensory abnormalities (e.g., pain, anosmia), and fatigue [3]. Among these, fatigue is one of the most disabling yet underrecognized symptoms, affecting up to 50% of patients and significantly impairing daily functioning and QoL [4,5]. Unlike general tiredness, PD-related fatigue is characterized by an overwhelming and persistent sense of physical and mental exhaustion that is often independent of motor fluctuations or sleep disturbances.

Since its introduction, levodopa (LD) has remained the gold standard for treating motor symptoms in PD. Despite its efficacy, during the disease course, nearly all patients on chronic LD therapy eventually develop motor fluctuation, defined as predictable or unpredictable variations in symptom severity throughout the day and night [6]. To manage motor fluctuations, clinicians may use different strategies, such as increasing and fractioning LD doses during the day, associating dopamine agonists, or adding enzymatic inhibitors such as catechol-O-methyl transferase (COMT) and monoamine oxidase type B (MAO-B) inhibitors [7,8]. Among these, MAO-B inhibitors, including selegiline, rasagiline, and safinamide, are commonly employed in clinical practice to enhance dopaminergic activity and prolong LD efficacy.

Unlike motor symptoms, NMS generally shows limited or unpredictable responses to dopamine replacement therapies, posing a considerable challenge in PD management [2]. Given the profound impact of fatigue on patients’ QoL and the lack of effective treatments, current research is focused on identifying pharmacological strategies that extend beyond dopaminergic modulation. In this context, MAO-B inhibitors have gained interest for their potential to modulate non-dopaminergic pathways and mitigate fatigue, offering a promising therapeutic avenue that warrants further investigation.

## 2. Fatigue in Parkinson’s Disease

Fatigue is one of the most prevalent and disabling NMS in PD, affecting up to 58% of PD patients throughout the disease course [4]. As a result of fatigue, PD patients often present limitations on daily living and social activities [5]. Notably, one-third of PD patients report fatigue as their most disabling symptom [9,10], including motor symptoms in the early disease stage [11,12].

A key distinction in PD-related fatigue lies between “physical” and “mental” fatigue, along with their respective fatigabilities. Physical fatigue refers to the perceived effort required to perform usual activities such as manual labor, walking, jogging, running, or lifting, which depend on the skeletal muscles to exert force. Physical fatigability is the decreased muscle ability resulting from motor tasks involving force production [13]. Mental fatigue, on the other hand, is the perceived effort needed to maintain focus on tasks, while mental fatigability refers to an individual’s ability to sustain attention or concentration over time [14].

### 2.1. Pathophysiology of PD-Related Fatigue

Current understanding of PD-related fatigue pathophysiology stems from different research approaches, including behavioral studies in human and animal models examining attentional processes and responses to salient stimuli [15]; f-MRI studies investigating aberrant connectivity in key brain regions such as the sensorimotor area, default mode network, and supplementary motor area [16,17]; and PET and pharmacological studies exploring the contribution of neurotransmitters such as dopamine, serotonin, and glutamate on fatigue [18,19]. Despite these insights, the exact mechanism underlying fatigue in PD remain elusive. Several hypotheses suggest a multifactorial etiology involving neuroinflammation [20], hypothalamic–pituitary–adrenal axis dysfunction [21], frontal striothalamocortical loops impairment [21], and autonomic dysregulation, including cardiac sympathetic denervation [22]. Recent evidence also suggests that orexinergic dysfunction may contribute to the development of fatigue in PD patients, with reduced orexin levels in the lateral hypothalamus leading to disturbances in sleep–wake regulation and energy balance, further exacerbating the multifactorial nature of this debilitating symptom [23]. Also, an imbalance among key neurotransmitter systems was suspected in the pathophysiology of fatigue. A more pronounced reduction in serotonin reuptake transporter (SERT) binding in the basal ganglia and thalamus is observed in PD patients with fatigue compared to those without fatigue [19,24]. While dopaminergic pathways appear to play a relatively minor role in fatigue pathogenesis [18], glutamatergic dysregulation is emerging as a key factor. Hyperactivity of glutamate-releasing neurons in the motor cortex—driven by the progressive loss of dopaminergic neurons in the substantia nigra pars compacta—induces subthalamic nucleus (STN) overactivity via both corticostriatal and corticosubthalamic pathways. This STN overactivity leads to excessive glutamate release, which overstimulates nigrothalamic GABAergic neurons, thereby sustaining motor symptoms through thalamic inhibition. Beyond its role in motor dysfunction, excessive glutamate activity is implicated in excitotoxicity, oxidative stress, mitochondrial dysfunction, and ultimately, neuronal death. Furthermore, glutamatergic hyperactivity contributes to neuroinflammation, a pivotal mechanism in PD progression [25]. Elevated glutamate levels can activate microglia, triggering the release of pro-inflammatory cytokines that amplify neuronal damage and perpetuate neurodegeneration [25]. In the context of PD-related fatigue, glutamate-driven neuroinflammation appears to be a central pathological factor. The resulting chronic inflammation and neuronal stress may accelerate disease progression and intensify NMS-like fatigue [20]. Therefore, targeting glutamatergic pathways to mitigate neuroinflammation may represent a promising therapeutic approach for alleviating fatigue and other related NMS in PD.

### 2.2. Fatigue Assessment

The profound impact of fatigue on daily functioning and overall well-being underscores the importance of its recognition and management. However, a universally accepted definition of fatigue is lacking, leading to its perception as a nonspecific and ubiquitous symptom. Moreover, fatigue exhibits considerable variability among patients, and unlike motor symptoms, no objective assessment methods are currently available. Fatigue frequently coexists with other NMS in PD patients, particularly sleep disturbances, apathy, and depression, which are commonly regarded as related phenomena [5]. Poor sleep quality is commonly reported as a trigger for fatigue in PD patients, with nocturnal sleep disturbances, including nocturia, REM sleep behavior disorder (RBD), and restless legs syndrome frequently associated with sleep fragmentation and daytime fatigue [26]. Despite these overlaps, each symptom has distinct biological underpinnings and responds differently to treatment [26]. Accordingly, a recent proposal of diagnostic criteria for PD-related fatigue specifies that it should not be considered a direct consequence of comorbid conditions such as depression or sleep disorders [25].

Fatigue perception is generally assessed through self-reported questionnaires, including the Fatigue Severity Scale (FSS), Parkinson’s Fatigue Scale (PFS), The Parkinson’s Disease Questionnaire (PDQ), Modified Fatigue Impact Scale (MFIS), Physical and Mental Fatigue Scales (PHYS-F, MENT-F), Multidimensional Fatigue Inventory (MdFI), and Visual Analog Scale Fatigue Scale (VAFS) [19,27]. Objective performance fatigability is commonly assessed by measuring changes in motor performance before and after a demanding task or during a sustained effort. Two main exercise paradigms are used: continuous maximal force tasks, which assess the decline in the ability to sustain a maximal voluntary contraction, and intermittent submaximal force tasks, which evaluate the ability to maintain repeated submaximal contractions over time [28]. To date, the limited studies available have failed to demonstrate correlations between objective measures of fatigability and subjective fatigue perception [28]. For instance, a recent study assessing objective physical performance fatigability using a MIDI keyboard in patients with PD found no significant correlation between measured fatigability and self-reported fatigue on the Modified Fatigue Impact Scale (MdFI) [28]. This highlights the complexity of fatigue in PD and the challenges associated with its assessment using objective measures alone. Selecting the most appropriate task paradigm or performance metric may enhance the ability to detect associations between fatigue perception and objective fatigability measures [5]. According to the Movement Disorder Society (MDS) Task Force [10], the Fatigue Severity Scale (FSS) is currently the only recommended tool for both screening and assessing fatigue severity in PD.

## 3. Monoamine-Oxidase Type B Inhibitors for PD Treatment

LD remains the cornerstone of PD treatment, but adjunctive therapies are often required to optimize symptom control and manage motor fluctuations. By reducing dopamine catabolism and increasing its synaptic availability, MAO-B inhibitors provide more continuous dopaminergic stimulation, making them particularly valuable in both the early and advanced stages of PD [29]. In early PD stages, selegiline and rasagiline are often used as monotherapy or in combination with low-dose LD [29]. Safinamide offers additional benefits in late-stage PD by modulating both dopaminergic and glutamatergic dysfunctions, providing a broader therapeutic effect [29]. Clinical trials confirm that adding MAO-B inhibitors to LD therapy decreases “OFF” periods and prolongs “ON” periods without worsening dyskinesias [30].

Side effects of MAO-B inhibitors include nausea, headache, insomnia, and dizziness, and rarely, more severe reactions like serotonin syndrome or hypertension [31]. When combined with serotonin-enhancing drugs (e.g., SSRIs), the risk of adverse effects increases, necessitating careful monitoring in polytherapy [32]. Additionally, MAO-B inhibitors can interact with foods high in tyramine, potentially causing hypertensive crises, especially with selegiline. This risk is lower with rasagiline and safinamide [32].

Beyond their motor benefits, emerging evidence suggests that MAO-B inhibitors may also exert positive effects on some NMS, including cognitive impairment, sleep disturbance, and fatigue [33]. However, their role in alleviating PD-related fatigue remains unclear. Given the significant impact of fatigue on patients’ QoL and the limited treatment options available, further investigation into the potential benefits of MAO-B inhibitors in this domain is warranted.

Interestingly, the use of MAO-B inhibitors extends beyond PD treatment. In fact, these drugs have been investigated for their role in other neurodegenerative and psychiatric conditions, such as Alzheimer’s disease and depression, but also migraine, epilepsy, Duchenne muscular dystrophy, multiple sclerosis, and ischemic brain injury [34]. These findings suggest that MAO-B inhibitors may modulate neurochemical pathways beyond dopaminergic systems, making them versatile therapeutic agents. This broad applicability underscores the need for further research to fully elucidate their potential across various conditions, including PD-related fatigue.

With this background, we conducted a narrative review to evaluate the role of MAO-B inhibitors in treating fatigue in PD patients, aiming to improve the literature and management of this distressing NMS (Table 1).

## 4. Methods

We conducted a narrative review following the Preferred Reporting Items for Systematic Reviews and Meta-analyses (PRISMA) guidelines, aiming to synthesize existing evidence on the role of MAO-B inhibitors in managing fatigue in PD. The literature search was performed on the PubMed database using the following keywords: “selegiline” OR “rasagiline” OR “safinamide” OR “MAO-B” AND “Fatigue” AND “Parkinson’s disease”. Only English-language articles published between January 1978 and August 2024 were considered. We included clinical trials and observational studies involving humans, as well as preclinical studies and case reports.

Inclusion criteria were: (a) patients with diagnosis of PD; (b) use of MAO-B inhibitors (selegiline, rasagiline, safinamide) for PD management; (c) assessment of fatigue as a primary or secondary endpoint using validated clinical scales or objective measures. Exclusion criteria included: (a) patients with diagnoses other than PD; (b) studies not investigating fatigue as an outcome or not reporting statistical significance of fatigue variations over time; and (c) reviews, meta-analyses, or expert opinions. No restrictions regarding the patient’s clinical/demographic features were applied.

Two independent reviewers (PP and SG) screened all relevant articles by title and abstract, followed by full-text assessment for eligibility. Discrepancies in the evaluations were discussed with the remaining authors. Additionally, the search was expanded to include references from the initially selected papers, and seminal studies were included regardless of publication date.

Given the heterogeneity of study designs and outcomes, a narrative synthesis with a thematic approach was employed to summarize findings. Studies were grouped based on intervention type, methodology, and reported effects on fatigue.

Since it is a narrative review, a formal risk of bias assessment using NHLBI or Cochrane tools was not performed. However, study quality was assessed considering key methodological aspects, including study design, sample size, fatigue assessment methods, and presence of control groups. Potential sources of bias, such as confounding factors and funding sources, were also taken into account in the interpretation of results.

## 5. Role of Monoamine-Oxidase Type B Inhibitors in PD-Related Fatigue

### 5.1. Selegiline

Selegiline is a selective, irreversible MAO-B inhibitor widely used in PD treatment. It is the R-enantiomer of deprenyl (phenyl-isopropyl-methyl-propargylamine) and primarily inhibits the MAO-B enzyme, thereby increasing dopamine levels in the central nervous system. Selegiline is rapidly absorbed from the gastrointestinal tract and efficiently crosses the blood-brain barrier. It undergoes extensive first-pass metabolism in the liver, producing active metabolites such as desmethylselegiline, methamphetamine, and amphetamine, which may contribute to its pharmacological effects [35].

Selegiline is typically administered at daily doses of 5–10 mg, either as monotherapy in the early stages of PD or in combination with LD and/or dopamine agonists in more advanced stages. It is available in both oral and transdermal formulations, with the latter designed to bypass first-pass metabolism and increase bioavailability.

Selegiline effectively alleviates motor symptoms in PD and may delay the need for higher doses of LD, thus reducing the risk of motor complications such as dyskinesia [35]. Selegiline also appears to be effective in addressing certain NMS. Gallazzi et al. [46] reported a significant reduction in excessive daytime sleepiness in PD patients, as measured by the Epworth Sleepiness Scale (ESS) and the Parkinson Disease Sleep Scale (PDSS), reflecting improved overall sleep quality. Notably, this effect may be partially attributed to its amphetamine-like metabolites, which have stimulant properties that could promote wakefulness and counteract fatigue.

The demonstration of selegiline’s effectiveness in improving fatigue arises from several preclinical studies. Contreras-Mora et al. [47] investigated its effect in alleviating motivational dysfunctions, which are believed to be related to fatigue and anergia. The study utilized a feeding task to examine the effects of selegiline on effort-related choice behavior. Selegiline at 6.0 mg/kg and 12.0 mg/kg significantly reversed the tetrabenazine-induced suppression of lever pressing in an animal model (*p* < 0.01). Lever pressing reflects a goal-directed effort to obtain a preferred food reward, and its suppression indicates reduced motivation. Additionally, intracranial injections of selegiline into the nucleus accumbens at doses of 2.0 and 4.0 μg also increased high-effort lever pressing and decreased chow consumption (*p* < 0.05), further supporting its role in enhancing motivation. Chow consumption refers to the intake of freely available standard food, requiring no effort; an increase typically reflects reduced willingness to exert effort for reward. The behavioral effects were correlated with increased extracellular dopamine in the nucleus accumbens, suggesting that the amphetamine-like metabolites of selegiline may contribute to these effects by promoting dopaminergic transmission.

Similarly, Yohn et al. [36] examined the impact of selegiline on effort-related motivation in rats using a feeding task. The study found that systemic administration of selegiline increased the selection of high-effort lever pressing, with a significant increase observed at a dose of 6.0 mg/kg (*p* < 0.05). At this dose, rats showed a marked increase in lever pressing on the progressive ratio schedule, while their chow intake decreased significantly (*p* < 0.05), indicating a shift towards more effortful behavior. Furthermore, microdialysis studies revealed that the 6.0 mg/kg dose of selegiline effectively increases high-effort lever pressing and significantly elevates extracellular dopamine levels in the nucleus accumbens (*p* < 0.05).

Although human studies on selegiline’s impact on fatigue are lacking, these findings suggest that selegiline may mitigate effort-related impairments associated with dopamine depletion, highlighting its potential in treating motivational dysfunction and fatigue in PD patients, particularly when these symptoms are resistant to standard dopaminergic therapies. By increasing dopamine availability in reward-related brain regions, selegiline may enhance motivation and alleviate fatigue in conditions like PD and depression.

### 5.2. Rasagiline

Rasagiline is a selective, irreversible MAO-B inhibitor with a therapeutic indication in PD patients either as monotherapy or in combination with LD [48,49,50,51], with a demonstrated significant efficacy in reducing motor fluctuations. In the PRESTO study [46], 1 mg/day of rasagiline significantly reduced daily “OFF” time by 1.85 h compared to 0.91 h with placebo (*p* < 0.001), while the ADAGIO study confirmed its effectiveness in reducing motor fluctuations when combined with LD [52].

Beyond its motor benefits, rasagiline has shown promise in improving NMS [33]. A double-blind, baseline-controlled trial investigated its effects on sleep quality using polysomnography (PSG) [53] and the PDSS-2. After eight weeks, rasagiline significantly improved sleep maintenance (+16.3%, *p* = 0.024), showed a trend toward better sleep efficiency (+12.1%, *p* = 0.097), and significantly reduced wake time after sleep onset (WASO) (*p* = 0.048). Additionally, it decreased the number of arousals and light sleep (N1 stage) (*p* = 0.019 and *p* = 0.036, respectively), suggesting enhanced sleep depth and continuity. However, despite these improvements in objective sleep measures, the study found no significant changes in subjective sleep quality measured by the PDSS-2 score and improvement in daytime sleepiness as measured by the ESS.

Evidence supporting rasagiline’s potential role in alleviating fatigue comes from multiple studies. A sub-study of ADAGIO by Stocchi et al. [37] evaluated rasagiline’s impact on fatigue in early PD. This 36-week, placebo-controlled, double-blind study included 1105 untreated PD patients divided into groups receiving rasagiline 1 mg/day (n = 270), rasagiline 2 mg/day (n = 277), or placebo (n = 558). Fatigue was assessed using the Parkinson Fatigue Scale (PFS), a patient-based, 16-item scale that evaluates the presence and severity of fatigue in PD. The PFS specifically captures the physical aspects of fatigue and its impact on activities of daily living while purposefully excluding mental fatigue components [54]. The PFS scores were compared at baseline and 36 weeks, showing a mean increase of 0.17 units in the placebo group, while rasagiline groups showed minimal changes (+0.03 units for 1 mg/day and −0.02 units for 2 mg/day, *p* < 0.01). The difference between rasagiline (both doses) and placebo was significant across various fatigue-related items, with *p*-values ranging from 0.009 to <0.0001. Moreover, rasagiline was associated with significantly less progression of fatigue symptoms in patients with early PD over 9 months compared to placebo. While the absolute changes in PFS scores were small, the consistent differences across multiple items suggest a meaningful clinical effect. The study cautions against interpreting rasagiline as a definitive treatment for fatigue, given the small effect size and the early stage of PD in the study population. However, this trial corroborates the findings of a study conducted by Schifitto et al. [18], which demonstrated that fatigue symptoms may manifest in the early stages of PD and tend to deteriorate over time in the absence of therapeutic intervention.

Further findings from the ADAGIO sub-study [38] focused on the combination of rasagiline and antidepressant in the improvement of depression, cognitive impairment, fatigue, and daytime sleepiness. Patients receiving both rasagiline and antidepressants experienced significantly less fatigue progression assessed using PFS when compared to the placebo group (*p* < 0.001), suggesting a potential benefit of rasagiline in managing fatigue symptoms in early PD. The study also assessed other NMS that could potentially influence fatigue, such as depression and sleep quality. Depression was evaluated using the Movement Disorder Society—Unified Parkinson’s Disease Rating Scale (MDS-UPDRS), which showed less worsening in depression scores in the rasagiline group compared to the placebo group (*p* = 0.048). Regarding sleep quality, daytime sleepiness scores revealed significantly less worsening in the rasagiline group compared to placebo (*p* = 0.006). However, the groups had no significant difference concerning overall sleep problems (*p* = 0.30).

Lim et al. conducted a double-blind, placebo-controlled pilot trial involving 30 randomized participants to receive either rasagiline (1 mg daily) or a placebo for 12 weeks [39]. The primary outcome measure for fatigue was the MFIS, a self-report measure that assesses the effects of fatigue on physical, cognitive, and psychosocial functioning. The study found a significant improvement in fatigue in the rasagiline group (12-point improvement) compared to the placebo group (8.5-point improvement) from baseline to week 12 (*p* = 0.003). Additionally, the FSS, which assesses the physical, social, and cognitive impact of fatigue, was used as a secondary measure, showing a significant 13-point improvement in the rasagiline group compared to a 3-point improvement in the placebo group (*p* = 0.027). In addition to these subjective assessments of fatigue, the study also employed objective measures of fatigability, both cognitive and physical. Cognitive fatigability was evaluated using the Paced Auditory Serial Addition Test (PASAT), which assesses sustained and divided attention as well as information processing speed. Fatigability was quantified as the decline in performance over time, specifically by calculating the ratio of correct responses during the first minute versus the second minute of the task. Motor fatigability was assessed with the finger tapping test, in which participants were instructed to tap as quickly as possible using the index finger on their more affected side for 60 s. The fatigability index was determined by comparing the number of taps in the first 30 s with the last 30 s. Both cognitive and motor fatigability improved more in the rasagiline group compared to the placebo group (*p* = 0.26 and *p* = 0.29, respectively). The study also explored other NMS, such as sleep quality and depression. While there were no significant changes in the PDSS scores, indicating no substantial improvement in subjective sleep quality, there was a notable trend in improvement in depression scores as measured by the Beck Depression Inventory-II (BDI-II) within the rasagiline group (*p* = 0.15) and a significant difference between groups (*p* = 0.018).

In addition to clinical trials specifically assessing the impact of rasagiline on fatigue, a report described three patients who developed a withdrawal syndrome after discontinuing rasagiline, presenting with psychiatric symptoms (depression, anxiety, panic attacks, agitation, dysphoria), generalized pain, autonomic dysfunction, and worsening fatigue [40]. This suggests rasagiline might play a role in the pathophysiology of fatigue, further supporting its potential benefit in PD-related fatigue management.

### 5.3. Safinamide

Safinamide is a reversible MAO inhibitor with high specificity for the MAO-B isoform [55], making it particularly effective in treating PD. It is administered orally in daily doses of 50 or 100 mg and demonstrates high bioavailability (80 to 92%). Unlike selegiline and rasagiline, safinamide is not metabolized by the cytochrome P450 enzyme system, reducing the risk of significant drug interactions. At a daily dose of 50 mg, safinamide achieves complete reversible inhibition of MAO-B activity, while a daily dose of 100 mg also inhibits glutamate release. Modulation of glutamatergic transmission has been observed in the basal ganglia and hippocampus and may help reduce glutamatergic overactivity [56,57]. As previously mentioned, glutamatergic transmission is implicated in striatal neurodegeneration and contributes to the motor fluctuations and dyskinesia that arise from chronic LD therapy [56]. Thus, alongside restoring nigrostriatal dopaminergic transmission, normalization of glutamatergic overactivity may alleviate PD symptoms and motor complications. Due to its dual dopaminergic and non-dopaminergic mechanism of action, safinamide, when used as an add-on to LD in fluctuating PD patients, effectively manages motor fluctuations by reducing “OFF” time and increasing “ON” time without worsening dyskinesia. Furthermore, it has demonstrated benefits in ameliorating various NMS.

The SAFINONMOTOR [41], an open-label prospective study, investigated the effects of safinamide as an add-on therapy on NMS in fluctuating PD patients, also exploring its impact on physical and mental fatigue. Fatigue improvement was assessed using VAFS-physical and VAFS-mental fatigue at multiple time points (baseline and after 1, 3, and 6 months). The Visual Analog Fatigue Scale (VAFS) is a self-reported measure that quantifies perceived physical and mental fatigue on a scale ranging from 0 (no fatigue) to 10 (maximum fatigue). VAFS-physical fatigue scores decreased by 12.9% (*p* = 0.293), while VAFS-mental fatigue scores decreased by 21.9% (*p* = 0.118) at 6 months from baseline. Fatigue was also evaluated using item 2 (sleep/fatigue) of the Non-Motor Symptoms Scale (NMSS), which showed a significant reduction (−35.8%, *p* = 0.002) at 6 months from baseline.

A longitudinal prospective study conducted by De Micco et al. [42] in a cohort of 20 fluctuating PD patients investigated whether safinamide 50 mg could improve non-motor, cognitive, and behavioral symptoms. The evaluation included PFS, performed before baseline and after 6 months of safinamide treatment. Results indicated a significant improvement in fatigue after 6 months of treatment (PFS respectively 2.85 ± 0.67 and 2.20 ± 1.07 at baseline and post-treatment; *p* = 0.02). Additionally, apathy, assessed using the Apathy Evaluation Scale (AES), showed a significant improvement after adding safinamide.

Another interesting study by Bianchi et al. [43] demonstrated a statistically significant improvement in fatigue following safinamide administration. This retrospective analysis included 20 fluctuating PD patients, evaluating NMS improvement. Patients were treated with safinamide starting at 50 mg/day for 15 days, then increased to 100 mg/day. The Physical and Mental Fatigue Scales (PHYS-F and MENT-F) were used to evaluate fatigue at baseline (T0) and after an average of 4.4 months of treatment (T1). Results showed a significant reduction in fatigue post-treatment, with the mean PHYS-F score decreasing from 10.8 at T0 to 6.7 after treatment (*p* = 0.03) and MENT-F decreasing from 9.8 at T0 to 6.1 post-treatment (*p* = 0.03).

Pauletti et al. [44] focused on the impact of safinamide on fatigue using FSS and PFS-16. This prospective, single-center observational pilot study involved 39 patients treated with safinamide for 24 weeks as add-on therapy, starting at 50 mg/day and increasing to 100 mg/day. The FFS and PFS-16 were administered at baseline (T0) and after 24 weeks of treatment (T1). Secondary variables, such as depression, QoL, motor, and NMS, were also assessed using validated questionnaires. After 24 weeks of safinamide treatment, they found a significant reduction in fatigue levels, with a significant decrease in FSS score from 5.1 ± 1.4 at T0 4.2 ± 1.6 at T1 (*p* = 0.001) and of PFS-16 score from 3.5 ± 0.9 at T0 to 3.2 ± 0.9 at T1 (*p* = 0.02). Furthermore, 46.2% of patients scored below the cut-off for fatigue presence on the FSS and 41% on the PFS-16, indicating that a substantial proportion of patients became “fatigue-free” after the treatment. The study also explored correlations between fatigue and other clinical variables, such as disease severity, duration, or dopaminergic drug treatment, but no significant correlations were found. However, there was a notable improvement in QoL in treatment-responsive patients, especially in mobility and activities of daily living.

Different results arise from the “VALE-SAFI” study [45], an observational single-center study of 60 patients with idiopathic PD on stable therapy with LD, evaluating the effect of safinamide on NMS and QoL. No significant changes in fatigue scores, assessed using the PDFS-16, were observed between baseline and follow-up. However, the item measuring “Life restricted by fatigue” showed minimal change (from 1.93 ± 1.30 at T0 to 1.73 ± 1.19 at T1, *p* = 0.451). Furthermore, while the NMSS total score showed a significant decrease from T0 to 6 months (*p* = 0.035), no change was observed in item 2 (sleep/fatigue). Despite this, safinamide had a notable impact on improving sleep quality, as evidenced by a significant decrease in the Pittsburgh Sleep Quality Index (PSQI) (*p* > 0.001) and overall QoL measured by the PDQ-39 (*p* = 0.012).

In conclusion, several studies support safinamide’s role in reducing PD-related fatigue. While results are mixed—with some studies showing significant improvements, whereas others do not—safinamide demonstrates potential in managing fatigue, possibly due to its effect on glutamate regulation and its ability to reduce motor fluctuations and apathy. These findings suggest that safinamide may offer a comprehensive approach to addressing fatigue in PD patients.

## 6. Discussion

MAO-B inhibitors are a well-established pharmacological option for treating motor symptoms and fluctuations in PD patients, with potential benefits for certain NMS. This review examined the role of MAO-B inhibitors in managing fatigue, a frequent and often debilitating NMS in PD. While LD remains the gold standard for managing motor symptoms, it does not effectively address fatigue, making this symptom a significant challenge for both patients and clinicians. Our review of the current literature suggests that MAO-B inhibitors may play a role in mitigating fatigue in PD, despite their different mechanisms of action.

Although the role of selegiline in fatigue management has not been definitively established in humans, its amphetamine-like metabolites suggest a potential benefit in reducing fatigue, as observed in the context of sleep disturbances in PD [35]. However, robust clinical evidence supporting this hypothesis remains limited and requires further confirmation through future clinical studies.

Several studies have investigated the potential benefits of rasagiline in reducing fatigue [37,38,39,54]. The relief of fatigue offered by rasagiline is often attributed to improvement in motor or other NMS, such as sleep disturbances [38,53], apathy, and depression [53] that frequently coexists with PD-related fatigue. However, from a phenomenological perspective, fatigue is distinct from sleepiness, lack of motivation, and depression, meaning it may persist despite effective treatment of these symptoms. Therefore, the observed improvement in fatigue likely follows a parallel pathway rather than merely reflecting the alleviation of these related phenomena. Supporting this hypothesis, in the study of Stocchi [37], depression did not impact the improvement in fatigue during rasagiline therapy. This study also found that PD patients in the rasagiline group were associated with less fatigue progression in early PD patients. Conversely, in the study by Lim et al. [39], the authors suggested that the observed improvement in fatigue in patients receiving rasagiline, compared to placebo, might be due to concurrent improvement in depression or motor function, as the physical component of the MFIS showed the most significant improvement in the rasagiline group. The extent to which improvement in depression, sleep, apathy, and motor symptoms contributes to fatigue reduction warrants investigation in future studies designed with adequate power and methodology.

While the role of safinamide in fatigue management is less established than that of rasagiline, several studies provide promising evidence, reporting improvement in fatigue in patients treated with safinamide. This effect may be linked to safinamide’s unique dual mechanism action as both an MAO-B inhibitor and a modulator of glutamate release, suggesting that its anti-glutamatergic properties may contribute to its effectiveness in managing this symptom. As with other MAO-B inhibitors, the benefit of safinamide in reducing fatigue has often been associated with improved sleep patterns [41], likely attributed to its modulation of glutamatergic pathways, given that poor sleep significantly contributes to both physical and mental fatigue. Additionally, safinamide’s known effect in reducing “OFF” periods could also play a role in fatigue improvement, as proposed by Hagell and supported by De Micco [42,58,59].

Although the role of MAO-B inhibitors in treating fatigue has been extensively explored in both clinical and preclinical studies, our findings align with a recent systematic review and meta-analysis by Siciliano et al. [60], which highlighted the lack of effective pharmacological treatments specifically targeting fatigue in PD. Similarly, a systematic review by Tsuboi et al. [33] confirmed the potential benefits of MAO-B inhibitors on selected NMS, such as depression, pain, and sleep disturbances, while emphasizing the scarcity of high-quality studies assessing their direct impact on fatigue. Nevertheless, the promising evidence supporting their role in fatigue management highlights the need for further research to delineate the specific role of MAO-B inhibitors in managing fatigue and to explore potential synergistic effects when combined with other therapies targeting.

## 7. Conclusions

In conclusion, while MAO-B inhibitors are well established in managing motor fluctuations, their role in alleviating fatigue in PD remains unclear. This review highlights the need for targeted research to clarify the mechanisms through which these drugs influence fatigue and to develop evidence-based treatment strategies tailored to this significant NMS. In particular, future studies should investigate the relationship between fatigue improvement and the alleviation of other NMS, as well as explore the potential role of glutamatergic modulation in fatigue management. Although preliminary findings suggest potential benefits, rigorous clinical trials are essential to confirm their efficacy, determine optimal dosing, and identify patient subgroups most likely to respond. Advancing this knowledge will be crucial for optimizing therapeutic approaches and improving the quality of life in PD.

## Figures and Tables

**Table 1:** Summary of key studies on the role of MAO-B inhibitors in fatigue in PD.

First Author; Year of Publication	Study Design	Model	Sub Jects (No.)	Follow-Up (Weeks)	Primary Endpoint	Fatigue Assessment	Results
Selegiline							
Contreras-Mora et al., 2018 [35]	Preclinical experimental study	Animal	Rats (24)	-	To investigate the effort-related motivational effects of tetrabenazine and their partial reversal by selegiline	Fixed Ratio 5 Chow Feeding Task	Selegiline partially reversed the motivational deficits induced by tetrabenazine. Animals treated with both tetrabenazine and selegiline exhibited improved performance in effort-based tasks compared to those receiving tetrabenazine alone
Yohn et al., 2018 [36]	Preclinical experimental study	Animal	Rats	-	To evaluate the effects of selegiline on effort-based decision-making in rats	Progressive Ratio Chow Feeding Task	Selegiline increased the selection of high-effort activities in rats. Animals treated with selegiline exhibited higher lever-pressing rates and increased breakpoint ratios in the progressive ratio/chow feeding choice task, indicating enhanced motivation to exert effort for a preferred reward
Rasagiline							
Stocchi et al., 2014 [37]	Sub-analysis of ADAGIO study: double-blind, placebo-controlled study, delayed-start trial of rasagiline in de novo PD	Human	De novo PD patients (n = 1105)	0, 36	To evaluate the effect of rasagiline in improving fatigue in early PD patients	PFS	Mean baseline PFS score was 2.2 ± 0.9 units. At 36 weeks, patients receiving placebo showed greater progression of symptoms (0.17 units) from baseline in PFS scores compared with the 1 mg/day (0.03 units) and 2 mg/day rasagiline groups (−0.02 units); the difference versus placebo was significant for both rasagiline groups (*p* < 0.01)
Smith et al., 2015 [38]	Post-hoc analysis of ADAGIO study: double-blind, placebo-controlled study, delayed-start trial of rasagiline in de novo PD	Human	De novo PD patients (n = 1174)	0, 36	To evaluate the effect of rasagiline on depression, cognition, sleep quality and fatigue in patients taking an antidepressant in PD patients	PFS	PFS scores revealed significantly less worsening in patients taking antidepressant and rasagiline compared with placebo (*p* < 0.001)
Lim et al., 2015 [39]	Double-blind, placebo-controlled study	Human	PD patients (n = 30)	0, 12	To evaluate whether rasagiline improved fatigue among PD patients	MFIS, FSS, MFI, PASAT, Finger tapping	Significant improvement of MFIS score was noted in the rasagiline group (12 points) as compared to placebo (8.5 points) from baseline to week 12 (*p* = 0.003). Change from baseline in FSS score was higher in the rasagiline group (13 points) compared to placebo (3 points improvement; *p* = 0.027). MFI (*p* = 0.04), PASAT score (*p* = 0.26) and Finger tapping (0.29) improved in the rasagiline group compared to placebo
Solla et al., 2022 [40]	Case report	Human	PD patients (n = 3)	-	-	-	Withdrawal syndrome after rasagiline suspension is characterized by prominent psychiatric disorders (depression, anxiety with panic attacks, dysphoria, and agitation) associated with generalized pain, autonomic manifestations and worsening of fatigue
Safinamide							
Santos García et al., 2021 [41]	SAFINONMOTOR: prospective open-label single-arm study	Human	Fluctuating PD patients (n = 44)	0, 4, 12, 24	To evaluate the change from baseline to the end of the observational period in the non-motor symptoms scale (NMSS) total score and subdomains in PD patients treated with safinamide	VAFS-physical fatigue, VAFS-mental fatigue, NMSS item 2 (sleep/fatigue)	Improvement was observed in VAFS-physical fatigue score (*p* = 0.293), VAFS-mental fatigue scores (*p* = 0.118), sleep/fatigue subdomain of NMSS (*p* = 0.002) at 6 months from baseline
De Micco et al., 2022 [42]	Longitudinal prospective study	Human	Fluctuating PD patients (n = 20)	0, 24	To evaluate whether safinamide 50 mg may improve non-motor, cognitive, and behavioral symptoms over a 6-month treatment period in fluctuating PD patients	PFS	Significant improvement in PFS after 6 months of treatment (*p* = 0.02)
Bianchi et al., 2018 [43]	Retrospective	Human	Fluctuating PD patients (n = 20)	17, 6	To evaluate the effect of safinamide on non-motor symptoms (NMS) in patients affected by idiopathic PD patients with motor fluctuations	PHYS-F, MENT-F	Significant reduction in fatigue post-treatment, with the mean PHYS-F score decreasing from 10.8 at T0 to 6.7 after treatment (*p* = 0.03) and MENT-F decreasing from 9.8 at T0 to 6.1 post-treatment (*p* = 0.03)
Pauletti et al., 2019 [44]	Prospective, single-center observational study	Human	Fluctuating PD patients (n = 39)	24	To test whether safinamide could represent an effective treatment of fatigue in fluctuating PD patients with fatigue before and after a 24-week treatment period with safinamide as add-on therapy	FSS, PFS-16	After 24 weeks of safinamide treatment a significant reduction in fatigue levels was found in FSS score (*p* = 0.001) and PFS-16 score (*p* = 0.02); 46.2% of patients scored below the cut-off for fatigue presence on the FSS and 41% on the PFS-16, indicating that a substantial proportion of patients became “fatigue-free” after the treatment
De Masi et al., 2022 [45]	VALE-SAFI study: observational, single-center study	Human	Fluctuating PD patients (n = 60)	24	To evaluate the effect of safinamide on NMS and QoL	PDFS-16, NMSS item 2 (sleep/fatigue)	No differences were found in PDFS-16 score between baseline and follow-up, but item 2 (life restricted by fatigue) showed minimal change (*p* = 0.451); no change was observed in item 2 (sleep/fatigue) of NMSS

PD: Parkinson’s disease; PFS: Parkinson’s Fatigue Scale; MFIS: Modified Fatigue Impact Scale; NMSS: Non-Motor Symptoms Scale; MFI: Multidimensional Fatigue Inventory; PASAT: hand grip strength-time; VAFS: Visual Analogue Fatigue Scale; PHYS-F: Physical Fatigue Scale; MENT-F: Mental Fatigue Scale; FSS: Fatigue Severity Scale; PDFS: Parkinson’s Disease Fatigue Scale; QoL: Quality of Life.

## Abbreviations

The following abbreviations are used in this manuscript:

PD	Parkinson’s disease
NMS	Non-motor symptom
QoL	Quality of life
SERT	Serotonin reuptake transporter
STN	Subthalamic nucleus
FSS	Fatigue Severity Scale
PFS	Parkinson’s Fatigue Scale
PDQ	Parkinson’s Disease Questionnaire
MFIS	Modified Fatigue Impact Scale
PHYS-F	Physical Fatigue Scale
MENT-F	Mental Fatigue Scale
NMSS	Non-Motor Symptom Scale
MdFI	Modified Fatigue Impact Scale
VAFS	Visual Analogue Fatigue Scale
MDS	Movement Disorder Society
LD	Levodopa
COMT	Catechol-O-methyl transferase
MAO-B	Monoamine oxidase type B
ESS	Epworth Sleepiness Scale
PDSS	Parkinson’s Disease Sleep Scale
PSG	Polysomnography
WASO	Wake after sleep onset
UPDRS	Unified Parkinson Disease Rating Scale
BDI	Beck Depression Inventory
PDFS	Parkinson’s Disease Fatigue Scale
PSQI	Pittsburgh Sleep Quality Index

## References

[1] Bloem B.R., Okun M.S., Klein C. (2021). Parkinson’s disease. Lancet.

[2] Poewe W. (2008). Non-motor symptoms in Parkinson’s disease. Eur. J. Neurol..

[3] Aubignat M., Tir M., Krystkowiak P. (2021). Les symptômes non-moteurs de la maladie de Parkinson de la physiopathologie au diagnostic précoce [Non-motor symptoms of Parkinson’s disease from pathophysiology to early diagnosis]. Rev. Med. Interne.

[4] Friedman J.H., Brown R.G., Comella C., Garber C.E., Krupp L.B., Lou J.-S., Marsh L., Nail L., Shulman L., Taylor C.B. (2007). Fatigue in Parkinson’s disease: A review. Mov. Disord..

[5] Herlofson K., Kluger B.M. (2017). Fatigue in Parkinson’s disease. J. Neurol. Sci..

[6] Widnell K. (2005). Pathophysiology of motor fluctuations in Parkinson’s disease. Mov. Disord..

[7] Fox S.H., Katzenschlager R., Lim S.Y., Barton B., de Bie R.M.A., Seppi K., Coelho M., Sampaio C., Movement Disorder Society Evidence-Based Medicine Committee (2018). International Parkinson and movement disorder society evidence-based medicine review: Update on treatments for the motor symptoms of Parkinson’s disease. Mov. Disord..

[8] Finberg J.P.M. (2019). Inhibitors of MAO-B and COMT: Their effects on brain dopamine levels and uses in Parkinson’s disease. J. Neural. Transm..

[9] Friedman J., Friedman H. (1993). Fatigue in Parkinson’s disease. Neurology.

[10] Friedman J.H., Alves G., Hagell P., Marinus J., Marsh L., Martinez-Martin P., Goetz C.G., Poewe W., Rascol O., Sampaio C. (2010). Fatigue rating scales critique and recommendations by the Movement Disorders Society task force on rating scales for Parkinson’s disease. Mov. Disord..

[11] Müller B., Assmus J., Herlofson K., Larsen J.P., Tysnes O.B. (2013). Importance of motor vs. non-motor symptoms for health-related quality of life in early Parkinson’s disease. Parkinsonism. Relat. Disord..

[12] Pfeiffer R.F. (2016). Non-motor symptoms in Parkinson’s disease. Parkinsonism. Relat. Disord..

[13] Kluger B.M., Krupp L.B., Enoka R.M. (2013). Fatigue and fatigability in neurologic illnesses: Proposal for a unified taxonomy. Neurology.

[14] Lou J.S. (2009). Physical and mental fatigue in Parkinson’s disease: Epidemiology, pathophysiology and treatment. Drugs Aging..

[15] Pauletti C., Mannarelli D., Locuratolo N., Currà A., Marinelli L., Fattapposta F. (2019). Central fatigue and attentional processing in Parkinson’s disease: An event-related potentials study. Clin. Neurophysiol..

[16] Tessitore A., Giordano A., De Micco R., Caiazzo G., Russo A., Cirillo M., Esposito F., Tedeschi G. (2016). Functional connectivity underpinnings of fatigue in “Drug-Naïve” patients with Parkinson’s disease. Mov. Disord..

[17] Siciliano M., De Micco R., Giordano A., Di Nardo F., Russo A., Caiazzo G., De Mase A., Cirillo M., Tedeschi G., Trojano L. (2020). Supplementary motor area functional connectivity in “drug-naïve” Parkinson’s disease patients with fatigue. J. Neural. Transm..

[18] Schifitto G., Friedman J.H., Oakes D., Shulman L., Comella C.L., Marek K., Fahn S., Parkinson Study Group ELLDOPA Investigators (2008). Fatigue in levodopa-naive subjects with Parkinson disease. Neurology.

[19] Pavese N., Metta V., Bose S.K., Chaudhuri K.R., Brooks D.J. (2010). Fatigue in Parkinson’s disease is linked to striatal and limbic serotonergic dysfunction. Brain.

[20] Lindqvist D., Hall S., Surova Y., Nielsen H.M., Janelidze S., Brundin L., Hansson O. (2013). Cerebrospinal fluid inflammatory markers in Parkinson’s disease—Association with depression, fatigue, and cognitive impairment. Brain Behav. Immun..

[21] Chaudhuri A., Behan P.O. (2004). Fatigue in neurological disorders. Lancet.

[22] Nakamura T., Hirayama M., Hara T., Hama T., Watanabe H., Sobue G. (2011). Does cardiovascular autonomic dysfunction contribute to fatigue in Parkinson’s disease?. Mov. Disord..

[23] Alster P., Madetko-Alster N., Otto-Ślusarczyk D., Migda A., Migda B., Struga M., Friedman A. (2023). Role of orexin in pathogenesis of neurodegenerative parkinsonisms. Neurol. Neurochir. Pol..

[24] Chaudhuri A., Behan P.O. (2000). Fatigue and basal ganglia. J. Neurol. Sci..

[25] Kluger B.M. (2017). Fatigue in Parkinson’s Disease. Int Rev Neurobiol..

[26] Diaconu S., Monescu V., Filip R., Marian L., Kakucs C., Murasan I., Chaudhuri K.R., Jianu D.C., Falup-Pecurariu C., Opritoiu B. (2024). The Impact of Fatigue on Sleep and Other Non-Motor Symptoms in Parkinson’s Disease. Brain Sci..

[27] Chen J., Zhou Y., Rao H., Liu J. (2024). Mental Fatigue in Parkinson’s Disease: Systematic Review and Evaluation of Self-Reported Fatigue Scales. Park. Dis..

[28] Lou J.-S., Kearns G., Benice T., Oken B., Sexton G., Nutt J. (2003). Levodopa improves physical fatigue in Parkinson’s disease: A double-blind, placebo-controlled, crossover study. Mov. Disord..

[29] Tan Y.Y., Jenner P., Chen S.D. (2022). Monoamine Oxidase-B Inhibitors for the Treatment of Parkinson’s Disease: Past, Present, and Future. J. Park. Dis..

[30] Chaudhuri K.R., Healy D.G., Schapira A.H., National Institute for Clinical Excellence (2006). Non-motor symptoms of Parkinson’s disease: Diagnosis and management. Lancet Neurol..

[31] Jost W.H. (2022). A critical appraisal of MAO-B inhibitors in the treatment of Parkinson’s disease. J. Neural. Transm..

[32] Aboukarr A., Giudice M. (2018). Interaction between Monoamine Oxidase B Inhibitors and Selective Serotonin Reuptake Inhibitors. Can. J. Hosp. Pharm..

[33] Tsuboi T., Satake Y., Hiraga K., Yokoi K., Hattori M., Suzuki M., Hara K., Ramirez-Zamora A., Okun M.S., Katsuno M. (2022). Effects of MAO-B inhibitors on non-motor symptoms and quality of life in Parkinson’s disease: A systematic review. NPJ Park. Dis..

[34] Alborghetti M., Bianchini E., De Carolis L., Galli S., Pontieri F.E., Rinaldi D. (2024). Type-B monoamine oxidase inhibitors in neurological diseases: Clinical applications based on preclinical findings. Neural Regen. Res..

[35] Magyar K. (2011). The pharmacology of selegiline. Int. Rev. Neurobiol..

[36] Yohn S.E., Reynolds S., Tripodi G., Correa M., Salamone J.D. (2018). The monoamine-oxidase B inhibitor deprenyl increases selection of high-effort activity in rats tested on a progressive ratio/chow feeding choice procedure: Implications for treating motivational dysfunctions. Behav. Brain Res..

[37] Stocchi F., ADAGIO investigators (2014). Benefits of treatment with rasagiline for fatigue symptoms in patients with early Parkinson’s disease. Eur. J. Neurol..

[38] Smith K.M., Eyal E., Weintraub D., ADAGIO Investigators (2015). Combined rasagiline and antidepressant use in Parkinson disease in the ADAGIO study: Effects on nonmotor symptoms and tolerability. JAMA Neurol..

[39] Lim T.T., Kluger B.M., Rodriguez R.L., Malaty I.A., Palacio R., Ojo O.O., Patel S., Gujrati Y., Nutter B., Swartz C. (2015). Rasagiline for the symptomatic treatment of fatigue in Parkinson’s disease. Mov. Disord..

[40] Solla P., Ercoli T., Masala C., Orofino G., Fadda L., Corda D.G., Zarbo I.R., Meloni M., Sechi E., Bagella C.F. (2022). Rasagiline Withdrawal Syndrome in Parkinson’s Disease. Brain Sci..

[41] Santos García D., Labandeira Guerra C., Yáñez Baña R., Cimas Hernando M.I., Cabo López I., Paz Gonález J.M., Alonso Losada M.G., González Palmás M.J., Martínez Miró C. (2021). Safinamide Improves Non-Motor Symptoms Burden in Parkinson’s Disease: An Open-Label Prospective Study. Brain Sci..

[42] De Micco R., Satolli S., Siciliano M., De Mase A., Giordano A., Tedeschi G., Tessitore A. (2022). Effects of safinamide on non-motor, cognitive, and behavioral symptoms in fluctuating Parkinson’s disease patients: A prospective longitudinal study. Neurol. Sci..

[43] Bianchi M.L.E., Riboldazzi G., Mauri M., Versino M. (2019). Efficacy of safinamide on non-motor symptoms in a cohort of patients affected by idiopathic Parkinson’s disease. Neurol. Sci..

[44] Pauletti C., Locuratolo N., Mannarelli D., Maffucci A., Petritis A., Menini E., Fattapposta F. (2023). Fatigue in fluctuating Parkinson’s disease patients: Possible impact of safinamide. J. Neural. Transm..

[45] De Masi C., Liguori C., Spanetta M., Fernandes M., Cerroni R., Garasto E., Pierantozzi M., Mercuri N.B., Stefani A. (2022). Non-motor symptoms burden in motor-fluctuating patients with Parkinson’s disease may be alleviated by safinamide: The VALE-SAFI study. J. Neural. Transm..

[46] Gallazzi M., Mauri M., Bianchi M.L., Riboldazzi G., Princiotta Cariddi L., Carimati F., Rebecchi V., Versino M. (2021). Selegiline reduces daytime sleepiness in patients with Parkinson’s disease. Brain Behav..

[47] Contreras-Mora H., Rowland M.A., Yohn S.E., Correa M., Salamone J.D. (2018). Partial reversal of the effort-related motivational effects of tetrabenazine with the MAO-B inhibitor deprenyl (selegiline): Implications for treating motivational dysfunctions. Pharmacol. Biochem. Behav..

[48] Parkinson Study Group (2005). A randomized placebo-controlled trial of rasagiline in levodopa-treated patients with Parkinson disease and motor fluctuations: The PRESTO study. Arch. Neurol..

[49] Elmer L.W. (2013). Rasagiline adjunct therapy in patients with Parkinson’s disease: Post hoc analyses of the PRESTO and LARGO tri-als. Park. Relat. Disord..

[50] Parkinson Study Group (2002). A controlled trial of rasagiline in early Parkinson disease: The TEMPO Study. Arch. Neurol..

[51] Jankovic J., Berkovich E., Eyal E., Tolosa E. (2014). Symptomatic efficacy of rasagiline monotherapy in early Parkinson’s disease: Post-hoc analyses from the ADAGIO trial. Park. Relat. Disord..

[52] Rascol O., Fitzer-Attas C.J., Hauser R., Jankovic J., Lang A., Langston J.W., Melamed E., Poewe W., Stocchi F., Tolosa E. (2011). A double-blind, delayed-start trial of rasagiline in Parkinson’s disease (the ADAGIO study): Prespecified and post-hoc analyses of the need for additional therapies, changes in UPDRS scores, and non-motor outcomes. Lancet Neurol..

[53] Schrempf W., Fauser M., Wienecke M., Brown S., Maaß A., Ossig C., Otto K., Brandt M.D., Löhle M., Schwanebeck U. (2018). Rasagiline improves polysomnographic sleep parameters in patients with Parkinson’s disease: A double-blind, baseline-controlled trial. Eur. J. Neurol..

[54] Martinez-Martin P., Wetmore J.B., Arbelo J.M., Catalán M.J., Valldeoriola F., Rodriguez-Blazquez C. (2019). Validation study of the Parkinson’s Fatigue Scale in advanced Parkinson’s disease. Patient Relat. Outcome Meas..

[55] Alborghetti M., Nicoletti F. (2019). Different Generations of Type-B Monoamine Oxidase Inhibitors in Parkinson’s Disease: From Bench to Bedside. Curr. Neuropharmacol..

[56] Salvati P., Maj R., Caccia C., Cervini M.A., Fornaretto M.G., Lamberti E., Pevarello P., Skeen G.A., White H.S., Wolf H.H. (1999). Biochemical and electrophysiological studies on the mechanism of action of PNU-151774E, a novel antiepileptic compound. J. Pharmacol. Exp. Ther..

[57] Morari M., Brugnoli A., Pisanò C.A., Novello S., Caccia C., Melloni E., Padoani G., Vailati S., Sardina M. (2018). Safinamide Differentially Modulates In Vivo Glutamate and GABA Release in the Rat Hippocampus and Basal Ganglia. J. Pharmacol. Exp. Ther..

[58] Hagell P., Brundin L. (2009). Towards an understanding of fatigue in Parkinson disease. J. Neurol. Neurosurg. Psychiatry.

[59] Liguori C., Stefani A., Ruffini R., Mercuri N.B., Pierantozzi M. (2018). Safinamide effect on sleep disturbances and daytime sleepiness in motor fluctuating Parkinson’s disease patients: A validated questionnaires-controlled study. Park. Relat. Disord..

[60] Siciliano M., Trojano L., Santangelo G., De Micco R., Tedeschi G., Tessitore A. (2018). Fatigue in Parkinson’s disease: A systematic review and meta-analysis. Mov. Disord..

